# Arundic acid attenuates retinal ganglion cell death by increasing glutamate/aspartate transporter expression in a model of normal tension glaucoma

**DOI:** 10.1038/cddis.2015.45

**Published:** 2015-03-19

**Authors:** M Yanagisawa, T Aida, T Takeda, K Namekata, T Harada, R Shinagawa, K Tanaka

**Affiliations:** 1 Laboratory of Molecular Neuroscience, Medical Research Institute, Tokyo Medical and Dental University, 1-5-45 Yushima, Bunkyo-ku, Tokyo 113-8510, Japan; 2 Visual Research Project, Tokyo Metropolitan Institute of Medical Science, 2-1-6 Kamikitazawa, Setagaya-ku, Tokyo 156-8506, Japan; 3 Discovery Research Laboratories I, Minase Research Institute, Ono Pharmaceutical Co. Ltd., 1-1, Sakurai 3-chome, Shimamoto-cho, Mishima-gun, Osaka 618-8585, Japan; 4 The Center for Brain Integration Research, Tokyo Medical and Dental University, 1-5-45 Yushima, Bunkyo-ku, Tokyo 113-8510, Japan; 5 JST, CREST, 7, Gobancho, Chiyoda-ku, Tokyo 102-0076, Japan

## Abstract

Glaucoma is the second leading cause of blindness worldwide and is characterized by gradual visual impairment owing to progressive loss of retinal ganglion cells (RGCs) and their axons. Glutamate excitotoxicity has been implicated as a mechanism of RGC death in glaucoma. Consistent with this claim, we previously reported that glutamate/aspartate transporter (GLAST)-deficient mice show optic nerve degeneration that is similar to that observed in glaucoma. Therefore, drugs that upregulate GLAST may be useful for neuroprotection in glaucoma. Although many compounds are known to increase the expression of another glial glutamate transporter, EAAT2/GLT1, few compounds are shown to increase GLAST expression. Arundic acid is a glial modulating agent that ameliorates delayed ischemic brain damage by attenuating increases in extracellular glutamate. We hypothesized that arundic acid neuroprotection involves upregulation of GLAST. To test this hypothesis, we examined the effect of arundic acid on GLAST expression and glutamate uptake. We found that arundic acid induces GLAST expression *in vitro* and *in vivo*. In addition, arundic acid treatment prevented RGC death by upregulating GLAST in heterozygous (*GLAST*^+/−^) mice. Furthermore, arundic acid stimulates the human GLAST ortholog, EAAT1, expression in human neuroglioblastoma cells. Thus, discovering compounds that can enhance EAAT1 expression and activity may be a novel strategy for therapeutic treatment of glaucoma.

Glaucoma affects nearly 70 million individuals worldwide and is one of the major causes of blindness in the developed world.^1^ The most common type of glaucoma is primary open angle glaucoma (POAG), which is characterized by gradual visual impairments owing to progressive loss of retinal ganglion cells (RGCs) and their axons.^2^ Although elevated intraocular pressure (IOP) is a risk factor for glaucoma, IOP elevation is not detected within a subset of POAG patients including those with normal tension glaucoma (NTG).^3^ Moreover, some patients continue to exhibit progressive loss of RGCs even after treatment to reduce IOP.^4^ These findings suggest that non-IOP-dependent factors may contribute to RGC degeneration. To better understand the pathogenesis of glaucoma and to develop improved therapies, it is necessary to discover these unidentified factors.

We previously reported that glutamate/aspartate transporter (GLAST)-deficient (*GLAST*^*−/−*^ and *GLAST*^+/−^) mice show spontaneous RGC death and glaucoma-like optic nerve degeneration without developing elevated IOP.^5, 6^ The GLAST protein is essential for maintaining the extracellular glutamate concentration below neurotoxic levels^7^ and for regulating glutathione levels in Müller glia by transporting glutamate,^8^ the substrate for glutathione synthesis, into the cells. Thus, GLAST deficiency results in RGC degeneration owing to excitotoxicity and oxidative stress. Moreover, previous work has shown that glutamate excitotoxicity and oxidative stress are involved in retinal damage in glaucoma.^9^ Furthermore, reportedly, the human GLAST ortholog excitatory amino-acid transporter 1 (EAAT1) is downregulated in the retinas of human patients with glaucoma.^10^ This result suggests that impairments in EAAT1 activity may be related to the pathogenesis of glaucoma. We previously reported that interleukin-1-dependent activation of GLAST increases glutamate uptake by Müller glia and protects RGCs from excitotoxicity.^11^ In addition, glial cell line-derived neurotrophic factor and neuturin-induced upregulation of GLAST in Müller cells is required to protect RGCs following optic nerve transection.^12^ Therefore, discovering new compounds that enhance EAAT1 activity may represent a novel strategy for therapeutic management of glaucoma.

In recent years, several groups have identified clinically useful drugs that activate EAAT2 transcription and translation.^13, 14, 15^ In the first study of this kind, Rothstein *et al.*^13^ reported that a *β*-lactam antibiotic, ceftriaxone, is neuroprotective *in vitro* and *in vivo* by increasing the expression of EAAT2. Although many compounds are known to increase EAAT2 expression, few drugs increase the expression of EAAT1/GLAST.^16^ Because of our specific interest in anti-glaucoma therapies, we wish to identify drugs that elevate EAAT1/GLAST expression and activity.

Arundic acid ((2R)-2-propyloctanoic acid, ONO-2506; Figure 1a) was originally discovered through screening for an agent to inhibit synthesis of S100*β* in astrocytes.^17^ A previous study showed that arundic acid administration markedly ameliorates brain damage in a transient middle cerebral artery occlusion rat model.^18^ These beneficial effects of arundic acid are associated with marked suppression of delayed extracellular glutamate accumulation in the peri-infarct areas.^19^ In searching for a possible mechanism of action, we hypothesized that arundic acid neuroprotection involves upregulation of EAAT1/GLAST.

To explore this hypothesis, we studied the effect of arundic acid on EAAT1/GLAST expression and glutamate uptake activity. These studies demonstrate that arundic acid can induce EAAT1/GLAST expression *in vitro* and *in vivo*. In addition, in GLAST heterozygous (*GLAST*^+/−^) mice, treatment with arundic acid prevents RGC death, mediated through upregulation of GLAST.

## Results

### Arundic acid increases glutamate uptake in mouse Müller cells by increasing GLAST expression

**Figure 1:**
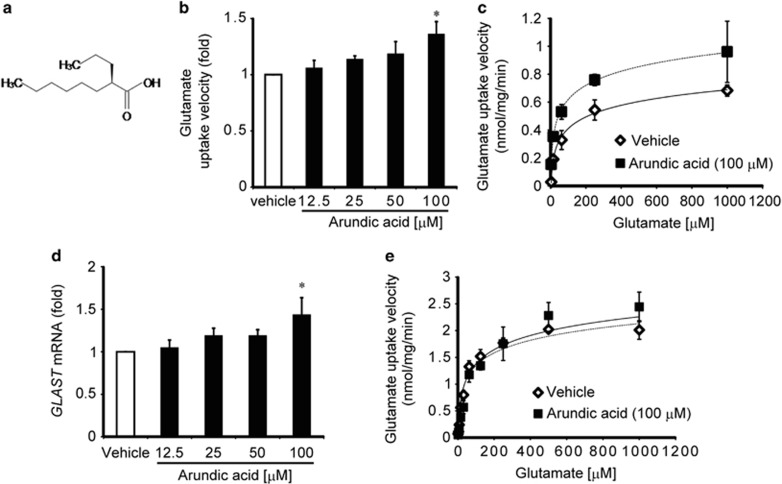
Arundic acid enhances glutamate uptake activity in Müller cells by an increase in the expression of *GLAST* mRNA. (**a**) Chemical structure of arundic acid. (**b**) In primary cultured Müller cells, glutamate transport is significantly increased after 14 days of treatment with 100 *μ*M arundic acid. *P<0.05 as determined by one-way ANOVA with Tukey–Kramer's *post hoc* analysis. (**c**) Representative transport kinetics saturation curves for l-[3,4-^3^H]-glutamate uptake activity in primary cultured Müller cells treated with 100 *μ*M arundic acid (closed square) or vehicle alone (open rhombus). Each data point corresponds to the mean±S.E.M. of three individual determinations. (**d**) Effects of arundic acid on *GLAST* mRNA expression in primary cultured Müller cells. *GLAST* mRNA expression is significantly increased following 100 *μ*M arundic acid treatment. *P<0.05 as determined by one-way ANOVA with Dunnett's *post hoc* analysis. (**e**) Transport kinetics analysis of glutamate uptake activity by EAAT1-expressing HEK293T cells following treatment with 100 *μ*M arundic acid (closed square) or vehicle alone (open rhombus). Data from three independent experiments generated mean values of 44.28±9.22 *μ*M for *K*_m_ and 2.04±0.51 nmol/mg/min for V_max_, in the absence of arundic acid, versus 54.96±18.57 *μ*M for *K*_m_ and 2.13±0.53 nmol/mg/min for *V*_max_, in the presence of arundic acid treatment. Thus, arundic acid had no effect on the kinetic properties of glutamate uptake by EAAT1-expressing HEK293T cells

To explore the postulated effects of arundic acid on glutamate uptake activity, first, we examined glutamate uptake in Müller cells that were prepared from retinas of C57BL/6 J mice and cultured for 14 days in the presence of 0–100 *μ*M arundic acid. The glutamate uptake velocity was significantly increased by 100 *μ*M arundic acid treatment (Figure 1b). Figure 1c shows the kinetic analysis of glutamate uptake in the presence and absence of 100 *μ*M arundic acid. The *V*_max_ value of cells treated with arundic acid was 1.48 times that of cells treated with vehicle alone (Student's *t*-test, *P*<0.05), whereas the *K*_m_ value was not significantly affected (42.15±18.98 *μ*M for vehicle alone *versus* 30.52±8.67 *μ*M for arundic acid). These results suggest that arundic acid increases glutamate uptake activity by increasing *V*_max_, without shifting the apparent glutamate affinity. One mechanism for increasing *V*_max_ is by increasing transporter expression. In Müller cells, GLAST is the most abundant glutamate transporter subtype.^20^ Thus, we examined whether arundic acid increases *GLAST* expression in Müller cells by quantitative real-time PCR (q RT-PCR). One hundred micromolar arundic acid significantly increased (*P*<0.05) endogenous *GLAST* mRNA expression in Müller cells (Figure 1d). The time course study revealed that arundic acid induced *GLAST* mRNA and protein expression as early as 24 h post-treatment (Supplementary Figures 1a and b). Because the transport process is driven by ion gradients, an arundic acid-mediated increase in glutamate uptake in Müller cells could be achieved indirectly by altering ion gradient across the plasma membrane. To explore this possibility, we examined the effect of arundic acid on glutamate uptake in HEK293T cells transfected with EAAT1 cDNA. In this system, arundic acid treatment did not alter the kinetic properties of EAAT1 (Figure 1e). Together, these results indicate that arundic acid treatment enhances glutamate uptake in Müller cells by increasing *GLAST* gene expression.

### Arundic acid selectively enhances glutamate uptake via GLAST in the retina

**Figure 2:**
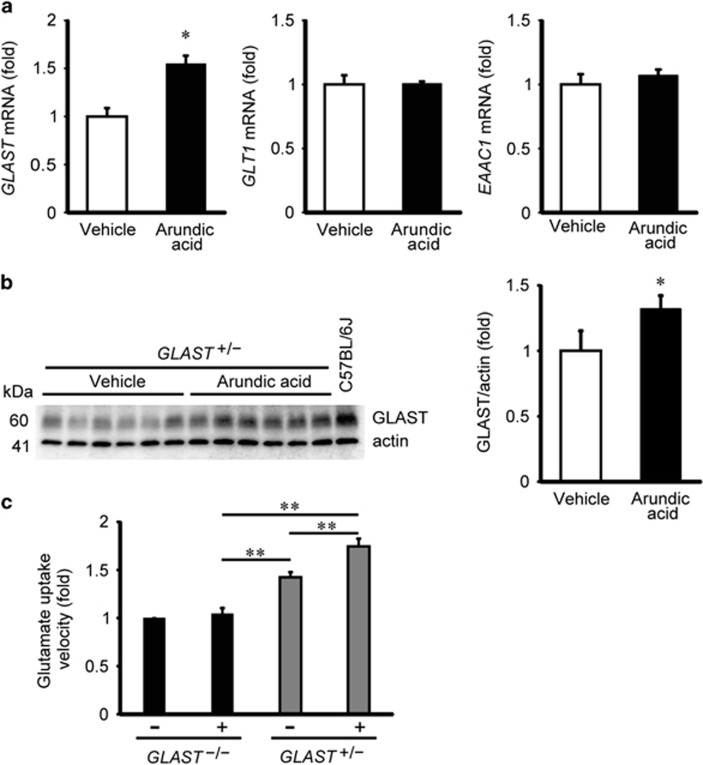
Arundic acid increases GLAST expression and transport activity in the mouse retina. (**a**) Effects of arundic acid treatment on *GLAST*, *GLT1* and *EAAC1* mRNA levels in the retina of *GLAST*^+/−^ mice. Arundic acid (10 mg/kg, given daily from P22 to P35) increased the *GLAST* mRNA level (*N*=6), whereas the mRNA levels of *GLT1* (*N*=6) and *EAAC1* (*N*=6) are unaffected. *P<0.05 as determined by a Student's *t*-test. (**b**) Arundic acid (10 mg/kg) increases GLAST protein expression in the retina of *GLAST*^+/−^ mice relative to vehicle-treated control mice (*N*=6). A representative western blot of GLAST protein expression is shown; the quantified data represent the mean±S.E.M. *P<0.05 as determined by a Student's *t*-test. (**c**) Effect of arundic acid on glutamate uptake activity in the retina of GLAST mutant mice. Relative glutamate uptake velocity was quantified from six independent experiments performed in duplicate for each data point. Data represent the mean±S.E.M. *P<0.05, **P<0.01 as determined by one-way ANOVA with Tukey–Kramer's *post hoc* analysis

To determine whether the effects of arundic acid are similar *in vivo*, we examined glutamate transporter expression in isolated mouse retinas treated with arundic acid. Three glutamate transporters are expressed around the synapses of RGCs in the plexiform layer, GLAST, GLT1 and excitatory amino-acid carrier 1 (EAAC1).^21^ We administered arundic acid or vehicle to *GLAST*^+/−^ mice during postnatal day (P) 22 to P35, and evaluated the glutamate transporter expression levels by qPCR. Arundic acid treatment increased *GLAST* mRNA expression in the retinas of *GLAST*^+/−^ mice, but did not alter *GLT1* or *EAAC1* mRNA levels (Figure 2a). Next, we asked whether arundic acid is capable of increasing GLAST protein expression in the retinas of *GLAST*^+/−^ mice. Using western blot analysis, we found that 14-day arundic acid treatment significantly increased endogenous GLAST protein expression (Figure 2b). To test whether the increases in GLAST mRNA and protein expression are accompanied by enhanced glutamate transport activity, we conducted l-[3,4-^3^H]-glutamate uptake assays in isolated retinas from *GLAST*^+/−^ and *GLAST*^*−/−*^ mice. Fourteen-day arundic acid treatment led to a 1.23-fold increase in glutamate uptake in the retinas of *GLAST*^+/−^ mice, compared with those treated with vehicle alone. We also observed that arundic acid increased GLAST protein expression and glutamate uptake activity in the retinas of wild-type mice (Supplementary Figures 2a and b). By contrast, arundic acid treatment did not affect glutamate uptake activity in the retinas of *GLAST*^*−/−*^ mice (Figure 2c). These results suggest that arundic acid treatment increases retinal glutamate uptake activity by selectively increasing GLAST expression *in vivo*.

### Arundic acid alleviates RGC loss by increasing GLAST expression in GLAST heterozygous mice

**Figure 3:**
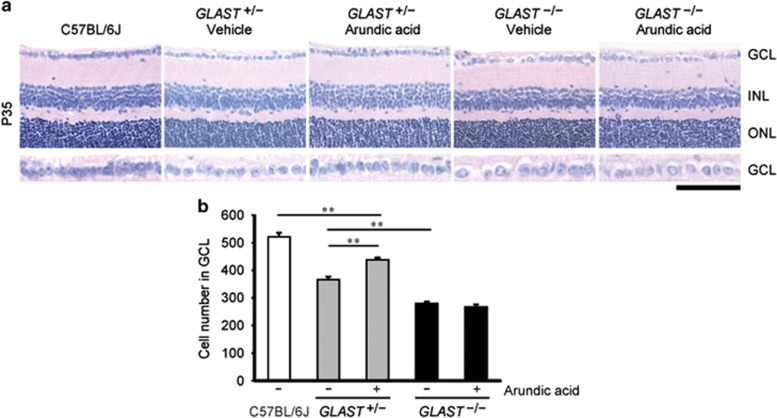
Arundic acid rescues RGC death in *GLAST*^+/−^ mice by increasing GLAST expression. (**a**) Hematoxylin and eosin-stained retinal sections from wild-type, *GLAST*^*+/*^^−^ and *GLAST*^*−/−*^ mice at P35, with or without arundic acid (10 mg/kg) treatment. The scale bar represents 100 *μ*m and 50 *μ*m in the upper and lower panels, respectively. GCL, ganglion cell layer; INL, inner nuclear layer; ONL, outer nuclear layer. (**b**) Quantitative analyses of the number of neurons in the GCL following arundic acid treatment. The numbers of neurons in the GCL were counted in retinal sections from one ora serrata through the optic nerve to the other ora serrata (*N*=6). The data represent the mean±S.E.M.. **P<0.01 as determined by one-way ANOVA with Tukey–Kramer's *post hoc* analysis

On the basis of the increased expression of GLAST described above, we hypothesized that arundic acid could be neuroprotective by protecting against RGC degeneration in GLAST-deficient mice. Chronic oral treatment of *GLAST*^+/−^ mice with arundic acid, starting at 22 days of age, lead to a significant prevention of RGC loss compared with vehicle-treated control *GLAST*^+/−^ mice (Figure 3). The number of cells in the ganglion cell layer (GCL) of *GLAST*^+/−^ mice subjected to arundic acid treatment was significantly increased (438±8 cells; *N*=6) relative to *GLAST*^+/−^ mice without arundic acid treatment (366±11 cells; *N*=6; Figures 3a and b). Neuroprotective effects of arundic acid cannot be seen with GLAST activation when studied in *GLAST*^*−/−*^ mice. Taken together, these results suggested that arundic acid attenuates RGC loss in *GLAST*^+/−^ mice by specifically facilitating GLAST expression.

### Arundic acid facilitates the endogenous EAAT1 expression in human neuroglioblastoma cells via activating EAAT1 promoter

**Figure 4:**
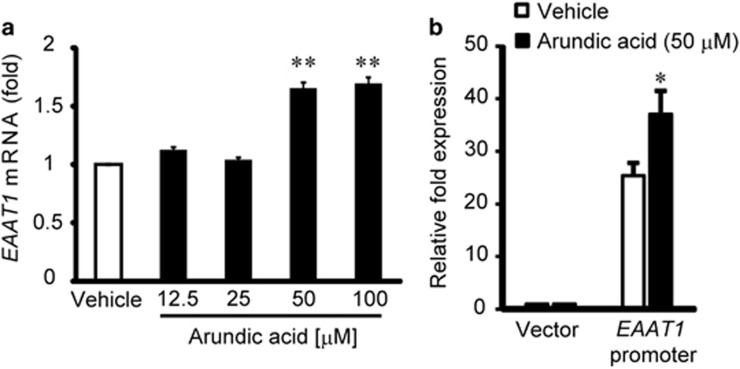
Arundic acid increases *EAAT1* mRNA and *EAAT1* promoter activity in human neuroglioblastoma H4 cells. (**a**) Arundic acid treatment resulted in an increase in *EAAT1* mRNA in the human H4 cell line, as evaluated by qPCR (*N*=4). Data represent the mean±S.E.M. **P<0.01 relative to control as determined by one-way ANOVA with Tukey–Kramer's *post hoc* analysis. (**b**) Arundic acid activates the *EAAT1* promoter. In human H4 cells transfected with the *EAAT1* promoter/luciferase reporter, 50 *μ*M arundic acid significantly induced *EAAT1* promoter activity (*N*=6). Data represent the mean±S.E.M. *P<0.05 as determined by a Student's *t*-test

Although arundic acid can enhance the expression of GLAST in retina of mice, it remains unclear whether arundic acid increases the expression of endogenous *EAAT1* in human cells. To study the effect of arundic acid on endogenous *EAAT1* expression in human glial cells, H4 human neuroglioblastoma cells^22^ were incubated with arundic acid for 9 days. *EAAT1* mRNA levels were quantified by qPCR. Treatment with 50 and 100 *μ*M arundic acid significantly increased *EAAT1* mRNA expression in H4 cells (Figure 4a). To better understand the mechanism of action, we examined the effect of arundic acid on the promoter activity of *EAAT1* in H4 cells. Previous studies showed that the full-length human *EAAT1* promoter compromised the 2.3 kb region immediately flanking the 5′-end of the human *EAAT1* gene^22^ and the 3′-UTR of the human *EAAT1* mediated the stimulatory influence of dbcAMP, epidermal growth factor, transforming growth factor *α* and pituitary adenylate cyclase-activating polypeptide on *EAAT1* expression.^23^ Thus, H4 cells were transfected with a reporter plasmid containing the full-length human *EAAT1* promoter in combination with the 3′-UTR of the human *EAAT1* at 6 days after 50 *μ*M arundic acid treatment. After 9 days of treatment of arundic acid, the cells were harvested and subjected to a luciferase reporter assay. Arundic acid significantly increased reporter gene activity of a construct containing the full-length human *EAAT1* promoter sequence as compared with vehicle-treated controls (Figure 4b). These data suggested that arundic acid can increase glutamate uptake in the human glial cells by activating the genetic promoter for *EAAT1*.

## Discussion

Our previous study showed that GLAST-deficient mice develop NTG-like phenotypes,^5^ which suggests that GLAST dysfunction may underlie or contribute to RGC loss in glaucoma patients. Importantly, deletion of *GLAST* in mice results in RGC degeneration without IOP elevation.^5^ Currently, IOP reduction is the only proven treatment of glaucoma. However, it should be noted that some glaucoma patients are still progressive despite sufficient IOP reduction.^4^ Thus, there is an urgent need for the discovery of alternative therapeutic approaches that are independent of IOP reduction and directed at preventing RGC loss. As glutamate excitotoxicity is involved in RGC loss in glaucoma,^9, 10, 24, 25, 26^ drugs capable of increasing GLAST may be useful neuroprotective compounds. In recent years, a number of groups have identified clinically useful drugs that elevate EAAT2 levels.^13, 14, 15^ However, there are few available drugs capable of increasing the expression of EAAT1/GLAST.^16^ Various preclinical investigations have demonstrated the manifold beneficial actions of arundic acid against neurological diseases, including ischemic stroke,^27^ Parkinson's disease,^28^ amyotrophic lateral sclerosis^29^ and Alzheimer's disease.^30^ Although a lot of studies have assessed the inhibitory effect of arundic acid on the production and release of S100*β* protein from glial cells in these diseases, no study has examined the effect of arundic acid on the expression and activity of glutamate transporters. Because glutamate excitotoxicity is involved in these neurological diseases,^31^ we hypothesized that the neuroprotective properties of arundic acid may, in part, involve the augmentation of EAAT1/GLAST. In this study, we demonstrated that arundic acid increases the EAAT/GLAST levels of the retina. This effect was specific to GLAST and did not alter the other subtypes of glutamate transporters, including the astroglial GLT1 and the neuronal EAAC1. It was reported that the neuroprotective action of arundic acid was mediated exclusively through the modulation of astrocytic function.^17^ In the brain, GLAST and GLT1 are mainly localized in astrocytes.^32^ However, in the retina, GLAST is expressed in Müller cells, whereas GLT1 is expressed only in a restricted set of neurons (mainly cone photoreceptors and cone bipolar cells).^20^ Thus, the selective upregulation of GLAST in the retina by arundic acid can be explained by the selective localization of GLAST in Müller cells, the major type of glial cells in the retina.

A previous study suggested that GLAST is also important for maintaining glutathione levels in Müller cells by transporting glutamate into the cells.^5^ Thus, GLAST dysfunction may lead to RGC degeneration through both excitotoxicity and oxidative stress. Arundic acid protected RGCs from oxidative and glutamate-induced injuries by increasing EAAT1/GLAST expression. Glutamate excitotoxicity and oxidative stress may contribute to retinal damage in various eye diseases, including retinal ischemia,^20^ glaucoma,^33, 34^ diabetic retinopathy^35^ and age-related macular degeneration.^36^ In addition, if neurons that contain high concentrations of glutamate are injured, then there can also be secondary excitotoxic damage. Therefore, augmentation of EAAT1/GLAST activity with arundic acid treatment may be a novel strategy for the management of glaucoma and other various forms of retinopathy. Furthermore, we observed that arundic acid enhances the glutamate uptake activity and expression of glial glutamate transporters, GLT1 and GLAST, in the cerebral cortex of wild-type mice (Supplementary Figures 3a and b). Arundic acid may be useful for the treatment of other neuropsychiatric diseases, such as ataxia,^37, 38^ migraine,^39^ schizophrenia^40, 41, 42^ and depression^43^ as glial glutamate transporter dysfunctions exist in these neuropsychiatric diseases. Arundic acid passed a phase 1 clinical trial,^27, 44^ so it does not cause toxicity in the central nervous system.

In addition, we demonstrated that arundic acid can enhance the EAAT1 expression in human neuroglioblastoma cells. The molecular mechanism of this enhancement appears to be activation of the genetic promoter for *EAAT1*, although the pathway for promoter activation is not known. Our studies provide potential novel neurotherapeutics for the management of glaucoma by modulating the EAAT1 activity via gene activation.

## Materials and Methods

### Mice and arundic acid administration

All experiments were performed in accordance with the ethical guidelines of the Institutional Animal Care and Use Committee of Tokyo Medical and Dental University. C57BL/6 J mice were purchased from CLEA Japan (Tokyo, Japan). *GLAST*^*+/–*^ and *GLAST*^*–/–*^ mice were previously described.^45^ All mice used in this study were backcrossed with C57BL/6 J mice for at least 10 generations. The daily oral administrations of arundic acid (10 mg/kg/day, Ono Pharmaceutical, Osaka, Japan)^30^ or corn oil (Sigma-Aldrich, St. Louis, MO, USA) to C57BL/6 J, *GLAST*^*+/–*^ and *GLAST*^*–/–*^ mice were performed from ages P22 to P35. At a dose of 10 mg/kg, orally administered arundic acid was shown to exhibit inhibitory actions on cerebral amyloidosis and gliosis in Altzheimer transgenic mice.^30^ The mice were killed immediately after the final administration and then their retinas were either processed for RGC counts, prepared for retinal RNA and protein extractions, or used for glutamate uptake assays.

### Cell culture, transfection and luciferase assays

The primary Müller cell cultures were prepared as previously described.^6, 46, 47^ The human neuroglioblastoma H4 cell line was purchased from American Type Tissue Collection (Manassas, VA, USA). Müller cells, H4 cells and HEK293T cells were grown in Dulbecco's modified Eagle's medium (Sigma-Aldrich) that contained 10% fetal bovine serum, 4.5 mg/ml d-glucose, 4 mM l-glutamine and 1 mM pyruvate at 37 °C in 10% CO_2_/90% O_2_. HEK293T cells were transfected with EAAT1 plasmid using GeneJuice Transfection Reagent (Merck Millipore, Billerica, MA, USA). One day after transfection, cells were plated onto 12-well plates at a density of 2.0 × 10^5^ cells per well and incubated for 1 day. Two days after transfection, the cells were subjected to a glutamate uptake assay. To study the effects of arundic acid on EAAT1 expression in H4 cells, the cells were incubated for 9 days in culture media that contained arundic acid (0, 12.5, 25, 50 or 100 *μ*M), and during which the media were changed every 2 days. To perform the luciferase promoter assay, after 6 days of 50 *μ*M arundic acid or DMSO treatment, H4 cells were transiently transfected with the pGL4.11[*luc2P*] (Promega, Madison, WI, USA), containing the full-length promoter region and 3′-UTR of *EAAT1*, and pGL4.73[*hRluc/SV40*] (Promega), using Lipofectamine3000 (Invitrogen, Carlsbad, CA, USA). After transfection, the cells were incubated with 50 *μ*M arundic acid or DMSO for three additional days. After 9 days of treatment, the cells were harvested with 1 × Passive Lysis Buffer (Promega). The luminescent signal from firefly luciferase and renilla luciferase was measured sequentially with a Lumat LB 9507 luminometer (Berthold Technologies, Bad Wildbad, Germany) using the Dual-Luciferase Reporter Assay System (Promega) according to the manufacturers' instructions. Firefly luciferase activity was normalized to renilla luciferase activity.

### DNA constructs

Full-length EAAT1 cDNA (OriGene, Rockville, MD, USA) was cloned in the mammalian expression vector pcDNA3.1 (Invitrogen). Genomic DNA was extracted from H4 cells using a DNeasy Blood & Tissue Kit (Qiagen, Valencia, CA, USA), and the 2.7 kilobase (kb) fragment of the *EAAT1* promoter and 2.1 kb fragment of *EAAT1* 3'UTR were amplified by PCR. Primer sets were designed as follows: *EAAT1* promoter forward 5′-GCTCGCTAGCCTCGAGGTAATCTCGAGTTCTTCAAACCAAT-3′ and reverse 5′-CCGGATTGCCAAGCTTGGTGGAAGATATCAAGCAGTAACG-3′ and *EAAT1* 3′-UTR forward 5′-AAATCGATAAGGATCCCGACAGTGAAACCAAGATGTAGAC-3′ and reverse 5′-AAGGGCATCGGTCTACAAGAATAACAACAACGTGCAAAGA-3′. The PCR product of the EAAT1 promoter was inserted between the *Xho*I and *Hin*dIII sites of pGL4.11[*luc2P*] and cloned using an In-Fusion HD Cloning Kit (Clontech, Mountain View, CA, USA), and then the PCR product of *EAAT1* 3′UTR was similarly cloned following insertion between the *Bam*HI and *Sal*I sites of pGL4.11[*luc2P*].

### Glutamate uptake assays

Two days after EAAT1 cDNA transfection, HEK293T cells were incubated at 37 °C for 12 min in assay buffer (137 mM NaCl, 5.4 mM KCl, 0.4 mM MgSO_4_, 0.5 mM MgCl_2_, 0.64 mM KH_2_PO_4_, 1.26 mM CaCl_2_, 5 mM HEPES (pH 7.5) and 5.5 mM d(+)-glucose) containing 1.0, 3.9, 15.6, 62.5, 250.0 or 1000.0 *μ*M of unlabeled l-glutamate. l-[3,4-^3^H] glutamate (50.6 Ci/mmol, PerkinElmer Life Science, Boston, MA, USA) was added to a final concentration of 0.05 *μ*M for an additional 20 min, following which the assay was terminated with two washes in ice-cold Na^+^-free assay buffer (NaCl was replaced by equimolar LiCl). Then the cells were immediately lysed with 0.1 N NaOH. Aliquots of the cell lysates were prepared for scintillation counting, whereas aliquots were used for the measurement of protein concentrations by a BCA kit (Sigma-Aldrich). The kinetic parameters, the Michaelis constant (*K*_m_) and the maximum uptake velocity (*V*_max_) were determined using Hanes–Woolf plot transformations. The effects of arundic acid on the kinetics of EAAT1 glutamate uptake activity were evaluated in cells following preincubation with arundic acid (100 *μ*M) and subsequent addition of l-[3, 4-^3^H] glutamate. In primary cultured Müller cells, the glutamate uptake assay was performed after 14 days of arundic acid treatment. Following 20 min of preincubation with assay buffer, glutamate uptake was terminated at 7 min by three washes in ice-cold Na^+^-free assay buffer. All results were from triplicate samples and were repeated in three separate experiments. To determine the glutamate uptake velocity of retinas from C57BL/6 J- and GLAST-deficient mice, both retinas were removed from one mouse and cut into eight pieces. Each set of four pieces was preincubated with either Na^+^-containing or Na^+^-free assay buffer and 100 *μ*M unlabeled glutamate for 20 min. l-[3,4-^3^H] glutamate was added to a final concentration of 0.05 *μ*M. After 7 min of incubation, the assay was terminated by washing three times with ice-cold Na^+^-free assay buffer. The glutamate transport velocity was calculated by subtracting the velocity in Na^+^-free assay buffer from that in Na^+^-containing assay buffer. All results were performed in duplicate samples and were repeated in six individual mice.

### Immunoblot analysis

Retinas and cultured Müller cells were homogenized in ice-cold 50 mM Tris-HCl (pH 7.5) containing 150 mM NaCl, 1% (v/v) Nonidet P-40, 0.25% (w/v) sodium deoxycholate, 1 mM EDTA, 1 mM phenylmethylsulfonyl fluoride, 1 mM NaVO_4_, 1 mM NaF and a proteinase inhibitor cocktail (Roche, Manheim, Germany). Samples were separated by sodium dodecyl sulfate polyacrylamide gel electrophoresis and then transferred onto polyvinylidene difluoride membranes (Merck Millipore). The following antibodies were used for immunoblotting: 1 *μ*g/ml of affinity purified anti-GLAST rabbit polyclonal^48^ and 10 ng/mL of anti-*β*-actin mouse monoclonal (C4; Santa Cruz Biotechnology, Santa Cruz, CA, USA) antibodies. After incubation with primary antibodies, the membrane was incubated with horseradish peroxidase-conjugated mouse or rabbit immunoglobulin G antibodies (diluted 1 : 10 000; Jackson ImmunoResearch Laboratories, Bar Harbor, ME, USA). Data were visualized using Luminata Forte Western HRP Substrate (Merck Millipore) and quantified by measuring the ratio of band intensities for GLAST relative to *β*-actin using Image Lab software (Bio-Rad Laboratories, Hercules, CA, USA).

### Quantitative RT-PCR experiments

Total mRNA was isolated from retinas and cells using TRIzol reagent (Invitrogen) and then reverse transcribed into cDNA using PrimeScript RT with gDNA Eraser (Takara Bio Inc. Siga, Japan). q RT-PCR was performed to amplify mouse *GLAST* (accession number: NM_148938.3), *GLT1* (accession number: NM_001077514.3), *EAAC1* (accession number: NM_009199.2) and *Rpp30* (the ortholog of human RNaseP, accession number: NM_019428.3). *EAAT1* and *RPPH1* (the H1 RNA subunit of the RNaseP enzyme complex, accession number: NR_002312.1) were amplified from human cells. The qPCR reactions were performed using a LightCycler 480 system II (Roche) with SYBR Premix ExTaq II (Takara Bio Inc.). The following primers were used: mouse *GLAST* forward 5′-GTCGCGGTGATAATGTGGTA-3′ and reverse 5′-AATCTTCCCTGCGATCAAGA-3′ mouse *GLT1* forward 5′-GGTCATCTTGGATGGAGGTC-3′ and reverse 5′-ATACTGGCTGCACCAATGC-3′ mouse *EAAC1* forward 5′-ACGTCACCCTGATCATTGCT-3′ and reverse 5′-GACGTTCACCATGGTCCTG-3′ mouse *Rpp30* forward 5′-TCCAGTGTGCAAGAAAGCTAAATG-3′ and reverse 5′-GGCAGTGCGTGGAGACTCA-3′ human *SLC1A3* forward 5′-TACCAAAGAGGAGGTTTGGC-3′ and reverse 5′-GGAGGGTCTCTTCTTTGCAC-3′ and human *RPPH1* forward 5′-AGCTGAGTGCGTCCTGTCACT-3′ and reverse 5′-TCTGGCCCTAGTCTCAGACCTT-3′. The qPCR experiments were conducted either three or four times, with every sample run in duplicate. The samples were normalized to the relative amplifications of mouse *Rpp30* and human *RPPH1.*

### Histological and morphometric analysis

Mice were killed at P35 and then their eyes were dissected and immersed in Davidson's fixative solution^24^ overnight at 4 °C. The fixed eyes were dehydrated in 70% ethanol for 3 days at 4 °C and embedded in paraffin wax. Embedded eyes were sectioned at a thickness of 7 *μ*m and stained with hematoxylin and eosin. The number of neurons in the GCL was counted from one ora serrata through the optic nerve to the other ora serrata in a blind manner. The average numbers of neurons in the GCL/eyes were obtained from three sections of each retina. Microscopic images were obtained using a Leica DM RA microscope (Leica, Wetzlar, Germany) with a HCX PLAN APO 40 × /0.75 PH2 objective (Leica) and a DFC 300 FX camera (Leica), and a Leica Application Suite (Leica).

### Statistical analyses

Values are expressed as the mean±S.E.M. Two-tailed Student's *t*-tests were used for two-sample comparisons, and one-way ANOVA tests were used for multiple comparisons followed by Tukey–Kramer's or Dunnett's *post hoc* tests for significance, in which *P*<0.05 was regarded as statistically significant.

## Abbreviations

GCL: ganglion cell layer
HEK: human embryonic kidney
INL: inner nuclear layer
IOP: intraocular pressure
NTG: normal tension glaucoma
ONL: outer nuclear layer
P: postnatal day
POAG: primary open angle glaucoma
RGC: retinal ganglion cell
