# Supplementary material for: Arundic acid attenuates retinal ganglion cell death by increasing glutamate/aspartate transporter expression in a model of normal tension glaucoma
Source: Cell Death Dis. 2015 Mar 19;6(3):e1693–. doi: 10.1038/cddis.2015.45 (PMC4385923; doi:10.1038/cddis.2015.45)
Supplement: Supplementary Information [file cddis201545x1.docx]

**Supplementary information**

**Arundic acid attenuates retinal ganglion cell death by increasing glutamate/aspartate transporter (GLAST) expression in a model of normal tension glaucoma**

Michiko Yanagisawa^1^, Tomomi Aida^1^, Takuya Takeda^1^, Kazuhiko Namekata^4^, Takayuki Harada^4^, Rika Shinagawa^5^, Kohichi Tanaka^1,2,3, *^

^1^Laboratory of Molecular Neuroscience, Medical Research Institute and ^2^The Center for Brain Integration Research, Tokyo Medical and Dental University, ^3^JST, CREST, ^4^Visual Research Project, Department of Sensory and Motor Systems, Tokyo Metropolitan Institute of Medical Science, ^5^Discovery Research Laboratories I, Minase Research Institute, Ono Pharmaceutical Co. Ltd., ^*^Corresponding author: Kohichi Tanaka, M.D. Ph.D.

**Supplementary Materials and Methods**

**Glutamate uptake assay of crude synaptosomes.** Arundic acid (10 mg/kg/day) or corn oil was orally administered to C57BL/6J mice from P22 to P35. Crude synaptosomes were prepared and glutamate uptake assay was performed as previously described^1^.

**Supplementary Figure legends**

**Supplementary Figure 1.** The time course of *GLAST* mRNA and protein expression in primary cultured Müller cells incubated with arundic acid. (a) The qPCR was performed at 12 hours, 24 hours, 3 days, 7 days and 14 days after arundic acid treatment. *GLAST* mRNA expression was significantly increased as early as 24 hours after treatment of 100 µM arundic acid compared with cells treated with vehicle. Each data point corresponds to the mean ± SEM of three individual determinations. * *P* < 0.05 and ** *P* < 0.01 as determined by Student’s t-test analysis. (b) A representative Western blot of GLAST protein expression in primary cultured Muller cells at 24 hours after treatment of 100 µM arundic acid is shown. GLAST protein expression was significantly increased as early as 24 hours after treatment of 100 µM arundic acid compared with cells treated with vehicle. Each data point corresponds to the mean ± SEM of three individual determinations. * *P* < 0.05 as determined by Student’s t-test.

**Supplementary Figure 2.** Arundic acid increases GLAST expression and transport activity in C57BL/6J mouse retina. (a) Arundic acid (10 mg/kg/day) or corn oil was orally administered to C57BL/6J mice from P22 to P35. Arundic acid increases GLAST protein expression in the retina of C57BL/6J mice relative to vehicle-treated control mice (N=6). A representative Western blot of GLAST protein expression is shown; the quantified data represent the mean ± SEM. * *P* < 0.05 as determined by a Student’s t-test. (b) Effect of arundic acid on glutamate uptake activity in C57BL/6J mouse retina. Relative glutamate uptake velocity was quantified from six independent experiments performed in duplicate for each data point. Data represent the mean ± SEM. * *P* < 0.05 as determined by Student’s t-test.

**Supplementary Figure 3.** Arundic acid increases GLT1 and GLAST expression and transport activity in the C57BL/6J mice cerebral cortex. (a) Representative transport kinetics saturation curves for L-[3,4-^3^H]-glutamate uptake activity in cortical crude synaptosomes of C57BL/6J mice administrated with arundic acid (closed square) or vehicle alone (open rhombus). Each data point corresponds to the mean ± SEM of six individual determinations. Although the Michaelis constant (K_m_) values determined under control conditions and in the presence of arundic acid were similar (28.22 ± 5.38 and 26.22 ± 3.43 µM, respectively), the maximum uptake velocity (V_max_) value was increased by arundic acid (from 2.15 ± 0.10 to 2.89 ± 0.09 nmol/mg/min, Student’s t test, *P* < 0.05). (b) Arundic acid increases GLT1 and GLAST protein expression in the cortex of C57BL/6J mice relative to vehicle-treated control mice (N=6). A representative Western blots of GLT1 and GLAST protein expression are shown; the quantified data represent the mean ± SEM. * *P* < 0.05 and ** *P* < 0.01 as determined by a Student’s t-test.

**Reference**

1. Tanaka K, Watase K, Manabe T, Yamada K, Watanabe M, Takahashi K*, et al.* Epilepsy and exacerbation of brain injury in mice lacking the glutamate transporter GLT-1. *Science* 1997; **276:** 1699-1702.
