# Supplementary figures and images for: Arundic acid attenuates retinal ganglion cell death by increasing glutamate/aspartate transporter expression in a model of normal tension glaucoma
Source: Cell Death Dis. 2015 Mar 19;6(3):e1693–. doi: 10.1038/cddis.2015.45 (PMC4385923; doi:10.1038/cddis.2015.45)

**Supplementary Figure 1.**

**a**

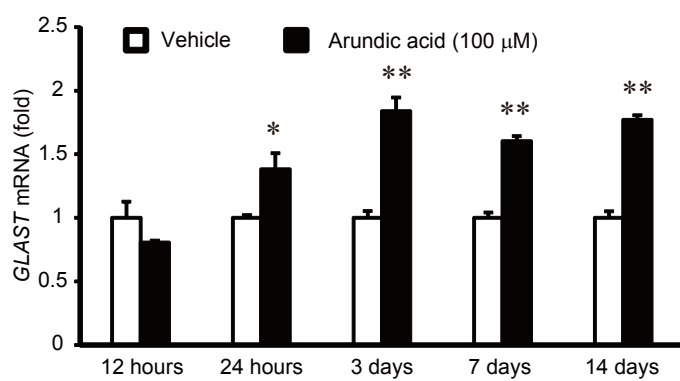

**b**

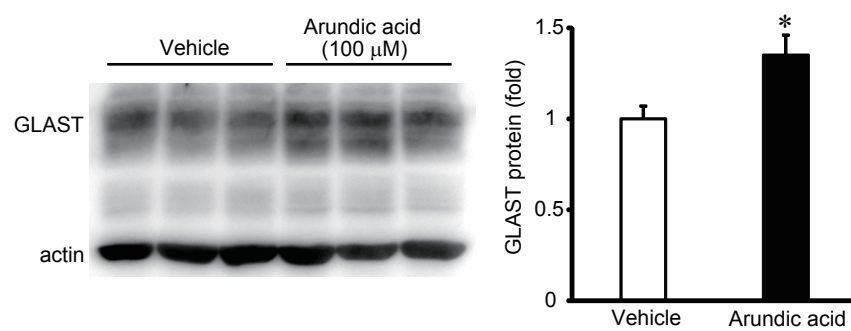

Supplement: Supplementary Figure 1 [file cddis201545x2.pdf]

Supplementary Figure 2.

**a**

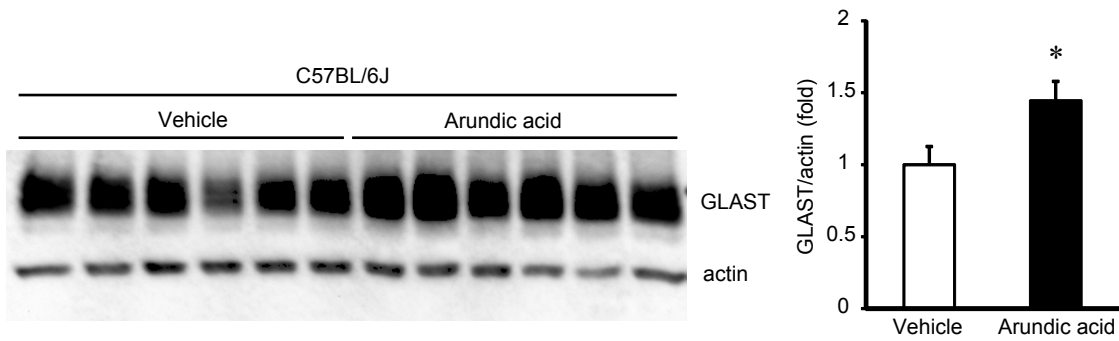

**b**

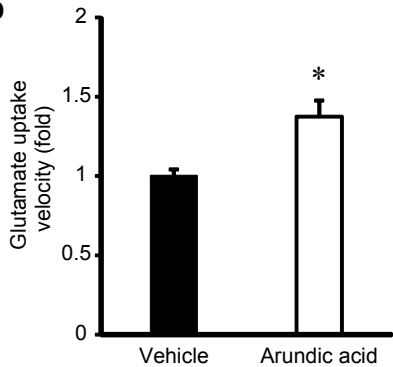

Supplement: Supplementary Figure 2 [file cddis201545x3.pdf]

**Supplementary Figure 3.**

**a**

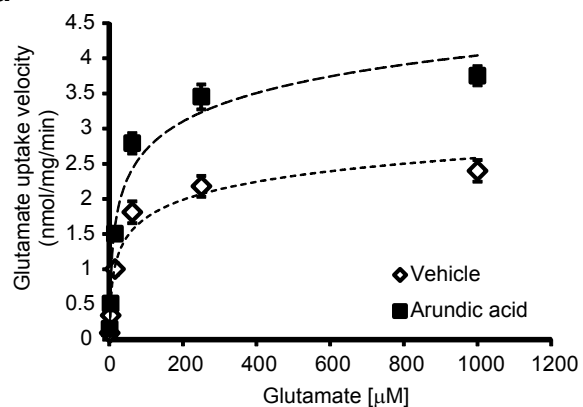

**b**

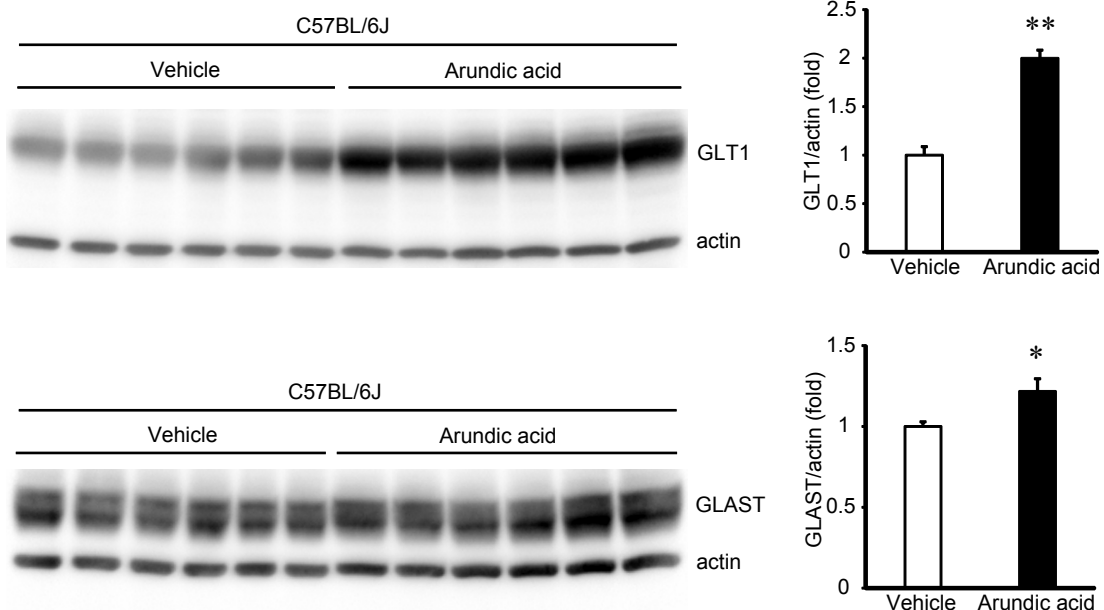

Supplement: Supplementary Figure 3 [file cddis201545x4.pdf]
